# Overexpression of KMT9α is associated with poor outcome in cholangiocarcinoma patients

**DOI:** 10.1007/s00432-025-06214-w

**Published:** 2025-05-13

**Authors:** Maximilian N. Kinzler, Eric Metzger, Rebecca Schulz, Katrin Bankov, Anna Ramos-Triguero, Falko Schulze, Steffen Gretser, Nada Abedin, Armin Wiegering, Stefan Zeuzem, Dirk Walter, Henning Reis, Roland Schüle, Peter J. Wild

**Affiliations:** 1 Goethe University Frankfurt, University Hospital Frankfurt, Medical Clinic 1, Theodor-Stern-Kai 7, 60590 Frankfurt am Main, Germany; 2 Klinik für Urologie und Zentrale Klinische Forschung, Klinikum der Albert-Ludwigs-Universität Freiburg, Freiburg, Germany; 3 Deutsches Konsortium für Translationale Krebsforschung, Freiburg, Germany; 4 Dr. Senckenberg Institutes of Pathology and Human Genetics, Goethe University Frankfurt, University Hospital Frankfurt, Frankfurt, Germany; 5 Charité – Universitätsmedizin Berlin, corporate member of Freie Universität Berlin and Humboldt-Universität zu Berlin, Department of Pediatric Oncology and Hematology, Augustenburger Platz 1, 13353 Berlin, Germany; 6 Department of Pediatric Oncology and Hematology, Charité— Universitätsmedizin Berlin, Berlin, Germany; 7 Department of General, Visceral, Transplant and Thoracic Surgery, Goethe University Frankfurt, University Hospital Frankfurt, Frankfurt, Germany; 8 Frankfurt Cancer Institute (FCI), Goethe University Frankfurt, Frankfurt, Germany; 9 Frankfurt Institute for Advanced Studies (FIAS), Frankfurt am Main, Germany

**Keywords:** Biomarker, Cholangiocarcinoma, Surgical oncology, Lysine methyltransferase 9, KMT9α

## Abstract

**Purpose:**

The newly discovered histone methyltransferase KMT9 serves as an epigenetic regulator of carcinogenesis in various cancer entities. For the first time, we investigated the presence of KMT9α in cholangiocarcinoma, the association with histologic subtypes, and its impact on survival.

**Methods:**

A tissue microarray cohort of all CCA patients who underwent surgical resection with curative intent between 08/2005 and 12/2021 at the University Hospital Frankfurt was immunohistochemically analyzed with the KMT9α antibody. For overall survival, Kaplan–Meier curves and Cox-regression analyses were performed.

**Results:**

In total, 174 patients were suitable for IHC analysis. Of the patients, 35.1% (*n* = 61) overexpressed KMT9α. Kaplan-Meier curves revealed a median OS of 34.75 months (95% CI = 20.23–49.27 months) for all CCA patients positive for KMT9α in comparison to 54.21 months (95% CI = 41.78–66.63 months) for patients lacking KMT9α overexpression (*p* = 0.004). Subtype analysis revealed strong differences in KMT9α expression. Multivariate Cox regression analysis identified KMT9α as an independent risk factor for shorter OS in CCA.

**Conclusion:**

This study demonstrates that a marked subset of CCA patients exhibit overexpression of KMT9α. These findings underscore the prognostic significance of KMT9α and reinforce its potential as a therapeutic target, consistent with its role in other cancer types.

**Supplementary Information:**

The online version contains supplementary material available at 10.1007/s00432-025-06214-w.

## Introduction

Cholangiocarcinoma (CCA) is a rare and highly heterogeneous malignancy that develops either from the intrahepatic biliary epithelium (iCCA) or from extrahepatic bile ducts (eCCA). While two main subtypes have emerged in the histopathological characterization of iCCA, i.e. small duct- (SD-iCCA) and large duct-type (LD-iCCA), a distinction can be made between perihilar (pCCA) and distal (dCCA) localization for eCCA (‘WHO Classification of Tumours 2019, 5th ed. Vol. 1. Digestive System Tumours, 2019. [Online]. Available: https://publications.iarc.fr/ Book-And-Report-Series/ Who-Classification-Of- Tumours/Digestive-System-Tumours-2019.’; Kinzler et al. 2022). The incidence of CCA is increasing while the prognosis remains unfavorable even after surgical resection (Groot Koerkamp and Fong 2014; Bertuccio et al. 2013).

In addition to genetic mutations, epigenetic changes also contribute to cancer development, shedding light on DNA methylation and histone modification as a focus of current cancer research (Yu et al. 2024; Hanahan and Weinberg 2011). Epigenetic modifications appear to be an important factor in the onset and development of CCA (O’Rourke et al. 2019; Banales et al. 2020; Chiang et al. 2015; Zhong et al. 2023). In line, several data indicate that DNA hypermethylation occurs in CCA in vitro and in vivo (Goeppert et al. 2014; Merino-Azpitarte et al. 2017).

Recently, a newly discovered histone lysine methyl transferase called lysine methyl transferase 9 (KMT9) was identified (Metzger et al. 2019). KMT9 monomethylates lysine 12 of histone H4 (H4K12me1) and is a heterodimeric enzyme consisting of KMT9α and KMT9β. In prostate cancer cells, KMT9 controls the expression of genes involved in the cell cycle and proliferation (Metzger et al. 2019). Baumert et al. revealed that KMT9 is also crucial for proliferation of lung cancer cells, while high levels of KMT9α correlate with poor patient survival (Baumert et al. 2020). In colorectal cancer, KMT9 regulates tumor cell proliferation and stemness while overexpression of KMT9α was associated with poor survival and metastasis in aggressive basal-like muscle-invasive bladder cancer (Berlin et al. 2022; Koll et al. 2023). Hence, KMT9 inhibition might be a therapeutic option for the treatment of several cancer entities. Accordingly, Wang et al. have recently developed a selective small-molecule KMT9 inhibitor (KMI169) with cellular activity in prostate and bladder cancer cells (Wang et al. 2024; Totonji et al. 2024).

Currently, no data exist on the expression of KMT9α in cholangiocarcinoma. Therefore, this study aimed to analyze the expression of KMT9α and its impact on the survival of CCA patients. Additionally, we sought to identify a potential subgroup of CCA patients who might benefit from future treatments targeting KMT9 inhibitors.

## Methods

### Study population

The study builds upon the patient population and data base of a previously reported tissue microarray (TMA) cohort of CCA patients that were surgically resected (R0, R1) at Frankfurt University Hospital between August 2005 and December 2021 (Kinzler et al. 2022a; Bankov et al. 2023). Histopathological confirmation was assessed by expert pathologists of the Dr Senckenberg Institutes of Pathology and Human Genetics, University Hospital Frankfurt. Small-duct and large-duct iCCA analyzed in this study were examined histomorphologically and immunohistochemically by an expert hepatobiliary pathologist. The tumors were classified into their respective subtypes based on criteria established in our previously published work (Kinzler et al. 2022). Clinical data, including gender, date of birth, tumor stage, tumor size, and comorbidities, were collected from electronic medical records. Tissue samples for this study were provided by the Senckenberg Biobank (SBB) as part of the Integrated Bio- and Databank Frankfurt (iBDF). Written informed consent was obtained from all patients, and the study received approval from the Institutional Review Boards of the UCT and the Ethics Committee of the University Hospital Frankfurt (project-number: UCT-27-2022_A2022).

### Immunohistochemistry (IHC) analysis

**Fig. 1:**
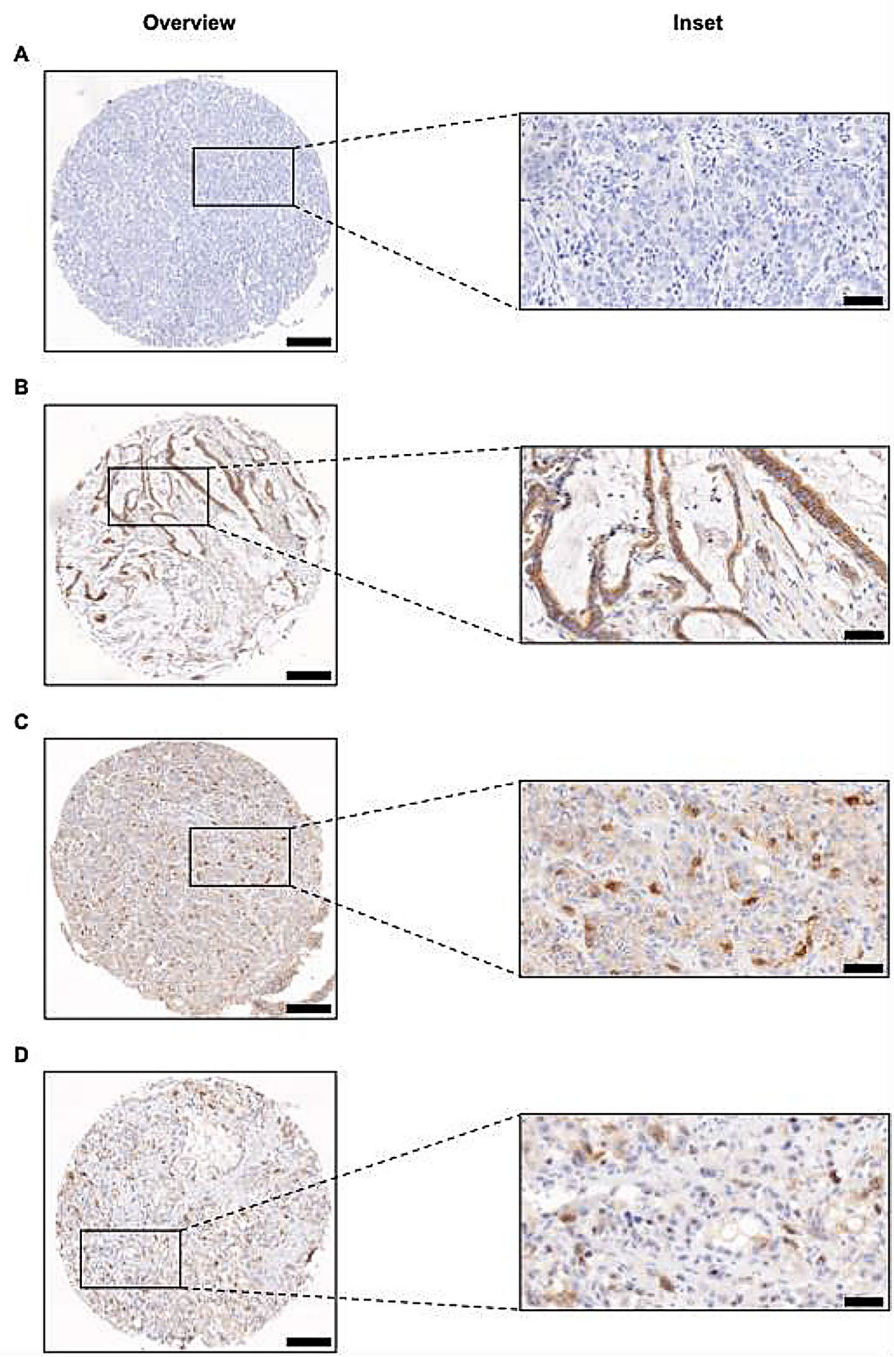
Representative images of KMT9α expression by immunohistochemistry. **A-D** Representative TMA cores of KMT9α immunohistochemistry in cholangiocarcinoma: negative (**A**), cytoplasmic only (**B**), nuclear (**C**) and nucleolar overexpression (**D**). Original magnification x8 for overview, x40 for inset, respectively. Scale bars = 200 μm for overview and 50 μm for inset, respectively

TMA construction was performed as previously reported (Kinzler et al. 2022). Staining of KMT9α (#27630; lot 20062017; dilution: 1:200; produced and provided by Schüle Lab) was conducted manually. Stained slides were scanned with the Pannoramic slide scanner (3DHISTECH, Budapest, Hungary). Staining of nuclear KMT9α in more than 1% of the tumor cells was considered as overexpression (≤ 1% = weak expression). Overexpression of KMT9a was assessed as positive, a weak expression or complete absence of KMT9 as negative. Overexpression of nucleolar KMT9α was observed in one case and statistically considered as nuclear positive case. Immunohistochemical evaluation of KMT9α was performed manually by two independent investigators. Figure 1 presents representative images illustrating various KMT9α staining patterns in CCA tissue, including negative, cytoplasmic-only, nuclear, and nucleolar staining.

### Statistical analysis

We compared baseline clinicopathological characteristics between patients with absence and presence of KMT9α expression. Categorial variables are presented as frequencies and percentages, continuous variables are shown as means with standard deviations. Categorial and continuous variables were compared using the Student’s t-test and chi-square test, respectively. Overall survival (OS) was defined as the time of onset of disease until death. Patients alive or lost to follow-up were treated as censored observation. Survival was compared using the log-rank test. Kaplan-Meier survival curves were generated to compare survival outcomes between CCA patients with and without nuclear KMT9α expression. To identify factors influencing patient survival, Cox regression analysis was conducted. Initially, univariate Cox regression was used to screen variables, and those with p-values < 0.05 were subsequently included in the multivariate Cox regression analysis. A significance level of *p* < 0.05 was applied for all analyses. Statistical analyses were conducted using SPSS version 27 (IBM; Armonk, BY, USA) statistical software and GraphPad Prism v.10.2.3.

## Results

### Clinical characteristics

In total, 174 patients with surgically resected CCA in our tertiary hospital were eligible for IHC analysis after TMA construction. Of the patients, 35.1% (*n* = 61) overexpressed KMT9α (2–20%, median 5%) while 64.9% (*n* = 113) were negative (Suppl. Figure 1). CCA patients with KMT9α overexpression were more likely to have larger tumor sizes (*p* = 0.005), higher pathological grading (*p* = 0.009), and abnormal serum lactate dehydrogenase levels (*p* = 0.015). In contrast, abnormal serum bilirubin levels were more frequently observed in KMT9α-negative patients (*p* = 0.005). No significant differences were found between KMT9α-positive and KMT9α-negative patients in other clinicopathological parameters (Table 1).

**Table 1:** Baseline characteristics

Characteristics	KMT9α pos. (*n* = 61) No. (%)	KMT9α neg. (*n* = 113) No. (%)	*p*-Value
Sex			0.82
Female	20 (32.8)	39 (34.5)	
Male	41(67.2)	74 (65.5)	
Age at initial diagnosis			0.197
mean, years, (range)	66.7 (41–86)	64.61 (38–86)	
CCA subtype			0.002
iCCA	47 (77)	58 (51.3)	
pCCA	8 (13.1)	29 (25.7)	
dCCA	6 (9.8)	26 (23)	
iCCA subtype			0.268
Small duct-type	31 (66)	44 (75.9)	
Large duct-type	16 (34)	14 (24.1)	
ECOG			0.633
0	40 (65.5)	78 (69)	
1	19 (31.1)	32 (28.3)	
2	2 (3.3)	3 (2.7)	
CA-19/9 (ng/ml)			0.091
< 37	20 (32.8)	44 (38.9)	
≥ 37	34 (55.7)	41 (36.3)	
n.a.	7 (11.5)	28 (24.8)	
Tumor size (cm)			0.005
≤ 5	27 (44.3)	75 (66.4)	
> 5	34 (55.7)	38 (33.6)	
Single Tumor			0.901
Yes	41 (67.2)	77 (68.1)	
No	20 (32.8)	36 (31.9)	
Pathological grade			0.009
Grade 1	0 (0)	3 (2.7)	
Grade 2	39 (63.9)	88 (77.9)	
Grade 3	22 (36.1)	22 (19.5)	
M status			0.163
Positive	6 (9.8)	5 (4.4)	
Negative	55 (90.2)	108 (95.6)	
N status			0.808
N0	41 (67.2)	70 (61.9)	
Pos. (regional)	16 (26.2)	39 (34.5)	
Pos. (distant)	4 (6.6)	4 (3.5)	
R status			0.484
R0	48 (78.7)	82 (72.6)	
R1	12 (19.7)	27 (23.9)	
Rx	1 (1.6)	4 (3.5)	
L status			0.567
L0	32 (52.5)	50 (44.2)	
L1	22 (36)	42 (37.2)	
Lx	7 (11.5)	21 (18.6)	
V status			0.72
V0	44 (72.1)	81 (71.7)	
V1	9 (14.8)	14 (12.4)	
Vx	8 (13.1)	18 (15.9)	
Pn status			0.506
Pn0	20 (32.8)	29 (25.7)	
Pn1	33 (54.1)	61 (54)	
Pnx	8 (13.1)	23 (20.4)	
Recurrence			0.985
Yes	26 (42.6)	48 (42.5)	
No	35 (57.4)	65 (57.5)	
PSC			0.339
Yes	1 (1.6)	5 (4.4)	
No	60 (98.4)	108 (95.6)	
Cholelithiasis			0.458
Yes	5 (8.2)	6 (5.3)	
No	56 (91.8)	107 (94.7)	
Viral hepatitis			0.268
Yes	3 (4.9)	11 (9.7)	
No	58 (95.1)	102 (90.3)	
Diabetes			0.890
Yes	14 (23)	27 (23.9)	
No	47 (77)	86 (76.1)	
Liver cirrhosis			0.173
Yes	1 (1.6)	7 (6.2)	
No	60 (98.4)	106 (93.8)	
LDH			0.015
<248	26 (42.6)	64 (56.6)	
≥248	20 (32.8)	19 (16.8)	
n.a.	15 (24.6)	30 (26.5)	
Bilirubin			0.005
<1.4	48 (78.7)	67 (59.3)	
≥1.4	11 (18)	44 (38.9)	
n.a.	2 (3.3)	2 (1.8)	

Abbreviations: CA-19/9 (Carbohydrate antigen 19-9), CCA (Cholangiocarcinoma), dCCA (distal Cholangiocarcinoma), ECOG (Eastern Cooperative Oncology Group), iCCA (intrahepatic Cholangiocarcinoma), LDH (Lactate dehydrogenase), n.a. (not available), No. (Number), pCCA (perihilar Cholangiocarcinoma), PSC (Primary sclerosing cholangitis)

### Expression of KMT9α in normal biliary epithelium and in cancerous tissue

**Fig. 2:**
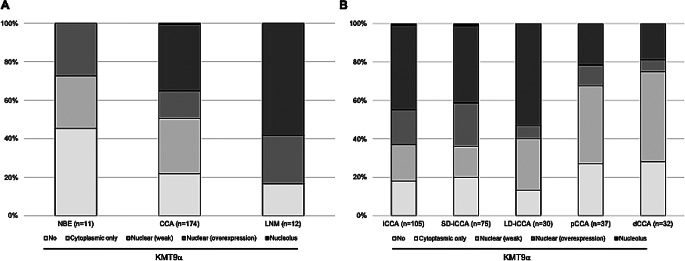
Distribution of KMT9α among normal biliary and cancerous tissue. **A/B (A)** Distribution of KMT9α in normal biliary epithelium, cholangiocarcinoma and lymph node metastasis. (**B**) Distribution of KMT9a among the different CCA subtypes. Abbreviations: CCA (Cholangiocarcinoma), dCCA (distal Cholangiocarcinoma), iCCA (intrahepatic Cholangiocarcinoma), LD-iCCA (Large duct-type intrahepatic Cholangiocarcinoma), LNM (Lymph node metastasis), NBE (Normal biliary epithelium), pCCA (perihilar Cholangiocarcinoma), SD-iCCA (Small duct-type intrahepatic Cholangiocarcinoma)

Given the substantial proportion of CCA patients with KMT9α overexpression, we further investigated potential differences between normal biliary epithelium and cancerous tissue. In normal biliary epithelium (*n* = 11), nuclear KMT9α overexpression was absent, which was significantly lower compared to CCA tissue (*p* = 0.016). Notably, KMT9α overexpression was higher in lymph node metastases (58.3%, *n* = 7) than in primary CCA tissue (35.1%, *n* = 61), although this difference did not reach statistical significance (*p* = 0.107) (Fig. 2A). Among CCA subtypes, KMT9α overexpression was significantly higher in iCCA compared to pCCA (*p* = 0.013) and dCCA (*p* = 0.008). However, no significant differences were observed between SD-iCCA and LD-iCCA (*p* = 0.268) or between pCCA and dCCA (*p* = 0.771). Remarkably, KMT9α overexpression was significantly increased in LD-iCCA compared to pCCA (*p* = 0.007) and dCCA (*p* = 0.004), respectively. Cellular distribution of KMT9α among the different CCA subtypes is depicted in Fig. 2B.

### Impact of KMT9α overexpression on overall survival

**Fig. 3:**
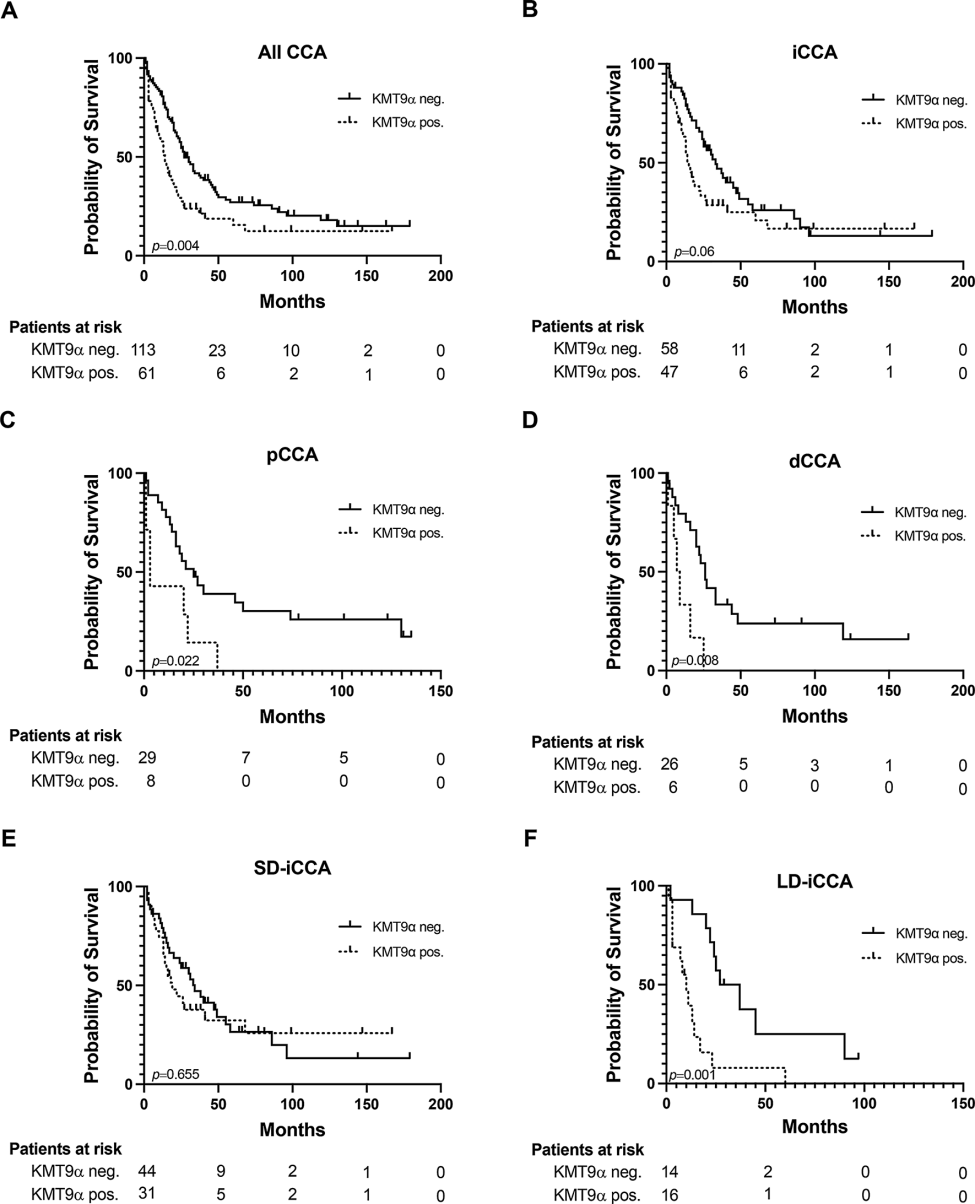
Kaplan-Meier curves for presence- and absence of KMT9α overexpression. **A-F** Overall survival assessed for presence- and absence of KMT9α overexpression in patients with CCA in general (**A**), iCCA (**B**), pCCA (**C**), dCCA (**D**), SD-iCCA (**E**) and LD-iCCA (**F**). Date of last follow-up was treated as censored observation. Abbreviations: CCA (Cholangiocarcinoma), dCCA (distal Cholangiocarcinoma), iCCA (intrahepatic Cholangiocarcinoma), LD-iCCA (Large duct-type intrahepatic Cholangiocarcinoma), pCCA (perihilar Cholangiocarcinoma), SD-iCCA (Small duct-type intrahepatic Cholangiocarcinoma)

Next, we aimed to assess the potential impact of KMT9α overexpression on overall survival (OS) in our cohort. Kaplan-Meier curves revealed a median OS of 34.75 months (95% CI = 20.23–49.27 months) for all CCA patients positive for KMT9α in comparison to 54.21 months (95% CI = 41.78–66.63 months) for patients lacking KMT9α immunoreactivity (*p* = 0.004) (Fig. 3A). In iCCA, OS rates were 42.75 months (95% CI = 24.1–61.42) and 53.72 (95% CI = 36.57–70.86) for presence and absence of KMT9α overexpression, respectively (*p* = 0.06) (Fig. 3B). For pCCA patients with present and absent KMT9α, the median OS was 12.5 months (95% CI = 3.46–21.54) and 46.89 (95% CI = 27.63–66.16), respectively (*p* = 0.022) (Fig. 3C). Correspondingly, KMT9α expression was associated with decreased overall survival rates in patients with dCCA, with a median survival of 10.5 months (95% CI = 3.56–17.43), compared to 49.56 months (95% CI = 26.87–72.26) in patients without KMT9α overexpression (*p* = 0.008) (Fig. 3D). In SD-iCCA, OS rates were 57.74 months (95% CI = 32–83.48) and 54.22 (95% CI = 33.87–74.6) for presence and absence of KMT9α, respectively (*p* = 0.655) (Fig. 3E). In contrast, OS rates were significantly reduced in LD-iCCA patients with KMT9α overexpression, with a median survival of 13.47 months (95% CI = 5.25–21.7), compared to 42.17 months (95% CI = 23.97–60.37) in patients without KMT9α overexpression (*p* = 0.001) (Fig. 3F).

### KMT9α overexpression is an independent risk factor for OS

Next, we performed both univariate and multivariate Cox regression analysis to identify potential risk factors associated with poor outcome. Univariate cox regression analysis revealed that KMT9α overexpression is a significant risk factor for OS (HR = 1.7, 95% CI = 1.2–2.4, *p* = 0.005). Moreover, ECOG status 1 (HR = 2.3, 95% CI = 1.6–3.4, *p* = < 0.001), multiple tumors (HR = 2, 95% CI = 1.4–2.9, *p* = < 0.001), positive tumor marker (HR = 2.1, 95% CI = 1.4–3.2, *p* = < 0.001), M1 status (HR = 2.6, 95% CI = 1.4–4.9, *p* = 0.003), regional- (HR = 2.2, 95% CI = 1.5–3.3, *p* = < 0.001) and distant lymph node metastasis (HR = 3, 95% CI = 1.4–6.4, *p* = < 0.001), R1 status (HR = 1.6, 95% CI = 1.1–2.4, *p* = 0.019), L1 status (HR = 1.7, 95% CI = 1.2–2.5, *p* = 0.004), V1 status (HR = 1.7, 95% CI = 1.1–2.8, *p* = 0.024), pathological grade 3 (HR = 4.4, 95% CI = 1.1–18.4, *p* = 0.041) and elevated serum bilirubin (HR = 1.7, 95% CI = 1.2–2.4, *p* = 0.004) also served as significant risk factors in univariate analysis (Table 2). Multivariate analysis revealed that overexpression of KMT9α (HR = 2.2, 95% CI = 1.4–3.7, *p* = 0.001), ECOG 1 (HR = 2.3, 95% CI = 1.4–3.9, *p* = 0.001), CA-19/9 (HR = 1.7, 95% CI = 1.1–2.7, *p* = 0.028), regional lymph node metastasis (HR = 2.2, 95% CI = 1.2–3.9, *p* = 0.007), V1 status (HR = 2.1, 95% CI = 1.1–4.3, *p* = 0.032) and elevated serum bilirubin (HR = 2.2, 95% CI = 1.3–3.7, *p* = 0.003) also serve as independent risk factors for OS for CCA patients (Table 2).

**Table 2:** Univariate and multivariate Cox regression analysis for overall survival

	Univariate analysis				Multivariate analysis		
Characteristics	HR	95% CI	*p*-Value		HR	95% CI	*p*-Value
Sex							
Female	ref						
Male	1.223	0.848–1.763	0.281				
CCA subtype							
iCCA	ref						
pCCA	1.192	0.778–1.825	0.419				
dCCA	1.172	0.749–1.833	0.487				
ECOG							
0	ref				ref		
1	2.287	1.554–3.365	< 0.001		2.327	1.398–3.871	0.001
2	2.846	1.153–7.076	0.23				
Tumor size (cm)							
≤ 5	ref						
> 5	1.091	0.77–1.545	0.624				
Single tumor							
Yes	ref				ref		
No	1.984	1.377–2.858	< 0.001		1.386	0.863–2.227	0.177
CA-19/9 (ng/ml)							
< 37	ref				ref		
≥ 37	2.134	1.434–3.175	< 0.001		1.701	1.059–2.734	0.028
M status							
Negative	ref				ref		
Positive	2.596	1.383–4.871	0.003		1.162	0.137–9.835	0.891
N status							
N0	ref				ref		
Yes (regional)	2.242	1.542–3.261	< 0.001		2.191	1.24–3.869	0.007
Yes (distant)	3.026	1.439–6.367	< 0.001		2.815	0.295–26.85	0.368
R status							
R0	ref				ref		
R1	1.601	1.081–2.372	0.019		1.139	0.648–2.001	0.651
Pn status							
Pn0	ref						
Pn1	1.392	0.929–2.086	0.109				
L status							
L0	ref				ref		
L1	1.719	1.184–2.496	0.004		0.783	0.44–1.392	0.404
V status							
V0	ref				ref		
V1	1.731	1.074–2.789	0.024		2.141	1.069–4.29	0.032
Pathological grade							
Grade 1	ref				ref		
Grade 2	1.503	0.369–6.117	0.569		0.966	0.121–7.712	0.974
Grade 3	4.415	1.059–18.41	0.041		1.584	0.189–13.24	0.671
KMT9α							
Neg.	ref				ref		
Pos.	1.673	1.169–2.395	0.005		2.244	1.362–3.695	0.001
Viral hepatitis							
No	ref						
Yes	0.568	0.278–1.163	0.122				
Liver cirrhosis							
No	ref						
Yes	0.91	0.371–2.232	0.837				
Diabetes							
No	ref						
Yes	1.116	0.747–1.669	0.591				
Cholelithiasis							
No	ref						
Yes	1.778	0.953–3.318	0.07				
PSC							
No	ref						
Yes	1.773	0.779–4.034	0.172				
LDH							
< 248	ref						
≥ 248	1.236	0.796–1.921	0.345				
Bilirubin							
< 1.4	ref				ref		
≥ 1.4	1.691	1.178–2.425	0.004		2.183	1.303–3.657	0.003

Abbreviations CA-19/9 (Carbohydrate antigen 19 − 9), CCA (Cholangiocarcinoma), CI (Confidence interval), dCCA (distal Cholangiocarcinoma), ECOG (Eastern Cooperative Oncology Group), HR (Hazard ratio), iCCA (intrahepatic Cholangiocarcinoma), LDH (Lactate dehydrogenase), pCCA (perihilar Cholangiocarcinoma), PSC (Primary sclerosing cholangitis)

## Discussion

The recently discovered histone methyltransferase KMT9 serves as an epigenetic regulator of carcinogenesis in various cancer entities (Metzger et al. 2019; Berlin et al. 2022; Baumert et al. 2020). Aim of the current study was to investigate the presence of KMT9α in cholangiocarcinoma, the association with histologic subtypes, and its impact on survival. To our knowledge, this study is the first to demonstrate that a significant subset of CCA patients exhibit KMT9α overexpression, emphasizing its prognostic value and potential as a therapeutic target, consistent with its role in other cancers.

In the present study, overexpression of KMT9α could be detected in a marked proportion of CCA patients (35.1%). In addition, we demonstrated a significantly reduced survival rate for these patients, with KMT9α overexpression serving as an independent risk factor for poor outcome. However, several aspects merit discussion as potential co-factors affecting survival in our study. Patients with KMT9α overexpression were more likely to have larger tumors, higher pathological grading and elevated levels of lactate dehydrogenase in serum, indicating a worse clinical course. In contrast, higher levels of bilirubin were more frequently observed in KMT9α-negative patients, potentially due to the larger proportion of p/dCCA in this cohort, as cholestasis and cholangitis are more common in eCCA (Yang and Zhang 2023).

A detailed subgroup analysis revealed that KMT9 overexpression predominantly occurred in iCCA, which had the highest positivity rate, followed by pCCA and dCCA. Moreover, within the group of iCCA, the large duct-type showed a higher frequency of KMT9α positivity compared to the small duct-type. It is known that both subtypes differ in underlying diseases, survival, response to chemotherapy, and molecular alterations (Kinzler et al. 2022a; Aishima and Oda 2015; Kendall et al. 2019; Gerber et al. 2022). While *IDH1* mutations and FGFR2 fusions occur mainly in the small duct-type (Kinzler et al. 2023; Arai et al. 2014), this study is the first to detect a potential therapeutically relevant biomarker occurring more frequently in the large duct-type. Remarkably, our data indicate that KMT9α overexpression had no impact on outcome in SD-iCCA, while survival rates were significantly reduced in the LD-iCCA counterparts. Furthermore, significant effects on survival were observed in p/dCCA, despite the moderate proportion of KMT9α-positive patients in this cohort. This is of high clinical interest, as LD-iCCA are generally mucin-secreting tubular adenocarcinomas resembling perihilar and distal CCA, and targeted options are most frequently lacking for these subtypes (Kendall et al. 2019).

Consistent with our findings, recent studies have demonstrated similar effects of KMT9 in various cancer entities. For instance, Metzger et al. analyzed a TMA cohort of patient-derived prostate tissue (Metzger et al. 2019). In line with our data, they found that nuclear KMT9α was absent in normal prostate tissue, while high levels of nuclear KMT9α increased with disease progression (Metzger et al. 2019). However, our results indicated even higher percentages of nuclear KMT9α in primary tumor tissue and lymph node metastasis of cholangiocarcinoma patients compared to the findings in prostate cancer (Metzger et al. 2019). Further studies also showed that KMT9α regulates proliferation and is linked to decreased survival in lung and colorectal cancer (Baumert et al. 2020; Berlin et al. 2022). However, comparability to our study is limited as their findings were based on non-/ small cell lung cancer cell lines, as well as mouse models and tumor organoids of colorectal cancer, whereas we focused on patient-derived tumor tissue. In contrast to our findings, no association between nuclear KMT9α and overall survival was detected in a TMA cohort of MIBC, although even better survival rates were observed in a subcohort of KMT9α-positive patients receiving adjuvant chemotherapy (Koll et al. 2023). In the same study, nucleolar expression of KMT9α was identified as an independent risk factor for poor survival in MIBC patients. Notably, we only detected one case with nucleolar expression of KMT9α, whereas Koll et al. reported that 17% (*n* = 23) of the patients with MIBC exhibited nucleolar KMT9α expression (Koll et al. 2023). Thus, we presume that nucleolar expression of KMT9α plays a minor role in CCA. Consequently, future studies are urgently needed to evaluate the effects of nuclear and nucleolar localization of KMT9α and its potential role as a relevant biomarker across various cancer types.

Aberrant DNA methylation and histone modification are well-known drivers of CCA development (Zhong et al. 2023). In this study, we demonstrated for the first time that overexpression of nuclear KMT9α is associated with lower survival. We therefore hypothesize that these effects may be linked to KMT9α-induced hypermethylation of lysine 12 of histone H4 (H4K12me1). Intriguingly, the histone lysine methyltransferase G9a, which catalyzes histone H3 lysine 9 (H3K9) dimethylation, was found to be upregulated in human CCA tissue and cells (Ma et al. 2020). Furthermore, survival analysis of TCGA data indicated that high G9a expression is associated with poor outcome in cholangiocarcinoma (Ma et al. 2020). Similarly, the histone methyltransferase EZH2, which catalyzes the methylation of lysine 27 on histone H3 (H3K27), has been shown to promote CCA development and progression in vitro, while corresponding TCGA data revealed worse survival rates in CCA patients expressing high EZH2 levels (Zhang et al. 2022). In conclusion, these preliminary data suggest that histone methyltransferases are promising targets for the therapeutic management of CCA, and that epigenetic markers may hold great clinical potential as diagnostic biomarkers in this entity.

To properly interpret the results of this study, it must be taken into account that our data are retrospective in nature and originate from a single center, which could lead to selection bias. Next, the sample size for p/dCCA was limited and generalizability may not be presumed for this subcohort. Importantly, we used treatment naïve tumor tissue of surgically resected specimen. However, future studies evaluating KMT9α and its potential role as a predictive marker for chemo-/ immunotherapy response in the palliative setting are warranted. This study integrates immunohistochemical, clinical, and survival data, offering important insights into the expression of KMT9α in a well-characterized CCA cohort. However, functional analyses to elucidate the observed effects are still pending and are essential for further exploration of KMT9α as a potential therapeutic target in CCA.

In conclusion, the present study is the first to provide a comprehensive overview of KMT9α expression in cholangiocarcinoma. Our data show that a relevant proportion of patients with CCA overexpress KMT9α, thereby impacting patient survival. Hence, future studies investigating the effect of KMT9α inhibition in vitro and in vivo are urgently needed to improve clinical management of patients suffering from CCA.

## Electronic supplementary material

Below is the link to the electronic supplementary material.

Supplementary Material 1

## Data Availability

The data that support the findings of this study are available from the corresponding author upon reasonable request.
