# Supplementary material for: Overexpression of KMT9α is associated with poor outcome in cholangiocarcinoma patients
Source: J Cancer Res Clin Oncol. 2025 May 13;151(5):161. doi: 10.1007/s00432-025-06214-w (PMC12069507; doi:10.1007/s00432-025-06214-w)
Supplement: Supplementary file 1 — Supplementary Material 1 [file 432_2025_6214_MOESM1_ESM.docx]

**Suppl. Figure 1.**
